# Spinal manipulation and mobilization forces delivered treating sciatica: a case report

**DOI:** 10.3389/fnint.2024.1356564

**Published:** 2024-07-08

**Authors:** Theodore B. Siciliano, Maruti R. Gudavalli, Ralph Kruse

**Affiliations:** ^1^Manahawkin Chiropractic Center, Manahawkin, NJ, United States; ^2^College of Chiropractic Medicine, Keiser University, West Palm Beach Campus, Fort Lauderdale, FL, United States

**Keywords:** force-based spinal manipulation, sciatica, Cox Technic flexion distraction decompression spinal manipulation, case report, low back pain

## Abstract

**Introduction:**

Accurately measuring the forces applied during spinal manipulation and its biomechanical effects on the spine are critically important in current research. This single case report discusses the potential benefit of accurately monitoring manipulative forces in treating low back pain with sciatica. The type of force-based spinal manipulation used to manage this case was Cox Technic flexion distraction decompression (CTFDD) spinal manipulation care, along with other ancillary modalities.

**Methods:**

The treatment plan, in this case, was primarily force-based CTFDD, equal-force bi-directional traction (EqFT), pre-modulated electrical muscle stimulation (EMS), infrared light therapy (ILT), and a home stretching and strengthening program.

**Clinical findings:**

Initially, the case study patient presented with complaints of left lumbar spine pain, which radiated into the left buttock, down the left leg, accompanied by an inability to dorsiflex the left foot. The patient was concerned with this condition as the left leg pain and left lower extremity motor deficit were having a profound effect on the patient’s ability to perform activities of daily living and work. The patient was recommended to undergo spinal decompression surgery, which the patient did not want, and elected to exhaust all alternative, non-surgical treatments first.

**Diagnosis, intervention, and outcomes:**

A diagnosis of sciatica with a sequestered disk fragment and left lower extremity motor deficit was rendered through objective physical examination results and a review of a lumbar MRI study. Past interventions included prescription medications, physical rehabilitation, chiropractic, pain management, and neurosurgical consultation. All past interventions prior to initiating CTFDD care provided minimal subjective and/or objective clinical improvement. This patient had a positive clinical outcome from a force-based CTFDD treatment plan along with other modalities consisting of pre-modulated EMS, ILT, and a home stretching and strengthening program.

**Conclusion:**

Force-based CTFDD spinal manipulation, along with other modalities consisting of pre-modulated EMS, ILT, and a home stretching and strengthening program, has been found to be an alternative, non-surgical treatment for discogenic sciatica. Continued research is needed on force-based CTFDD spinal manipulation to further evaluate the neurological and biomechanical effects of the forces and motion applied to the spine and determine health benefits for the treatment of low back pain.

## Introduction

Low back pain is the foremost musculoskeletal condition affecting people’s activities of daily living, causing lost wages and workplace disability. The estimated economic costs of low back pain in the United States are upward of $100 billion (Katz, 2006; Vos et al., 2012; Hoy et al., 2014). Low back pain with associated sciatica is commonly caused by mechanical compression from a herniated intervertebral disk on a lumbar spine nerve root (Meng et al., 2022). Sciatica can present with a neurological motor deficit of the lower extremity, manifesting as an inability to dorsiflex the foot (Cox, 2011). A patient diagnosed with discogenic sciatica with a lower extremity motor deficit may require interdisciplinary input, such as a neurosurgical consultation, as a progressive neurological deficit is one of the key indicators for surgical intervention (Sharma et al., 2012; Wielechowski et al., 2020).

The purpose of this case report is to discuss how measuring the forces and movements of a force-based Cox Technic flexion distraction decompression (CTFDD) spinal manipulation along with other modalities may be of clinical benefit. Furthermore, it discusses how CTFDD, along with other modalities, can be an alternative, non-surgical treatment for sciatica originating from an L4–L5 disk herniation.

## Methods

Force-based CTFDD is a low-velocity, variable-amplitude form of spinal mobilization. Research documents the biomechanical effects of CTFDD on the lumbar spine, including increased intervertebral disk space heights, decreased intradiscal pressure, and increased vertebral foraminal area (Cox, 2011; Gudavalli et al., 2022, 2023). CTFDD uses the Cox 8 Force Table (C8FT) that can simultaneously flex and distract the lumbar spine while applying equal bi-directional distraction forces. The distraction forces measured by the C8FT are cephalad hand force (HF) and caudalward Long y-Axis Force (LyAF), both displayed in pounds (Cox, 2011). The caudal section movements of the C8FT are lateral flexion and flexion angle, which are measured in degrees. The CTFDD data are collected by force transducers located in the C8FT, then imported to a computer and shown as force and motion graphs. The CTFDD force and motion data can then objectively guide CTFDD treatment parameters by monitoring the bi-directional manipulative forces as noted by patient tolerance and decreasing spinal resistance (Jun et al., 2020; Amjad et al., 2022). CTFDD spinal manipulation has two basic treatment protocols, protocol 1 and protocol 2. Prior to administering any CTFDD treatment protocols, the patient is always tested for tolerance to ensure treatment viability. Tolerance testing prior to CTFDD is important in ensuring a successful clinical outcome. Protocol 1 is for sciatica patients and consists of three sets of five cycles of equal cephalad HF and caudalward LyAF. Protocol 1 is primarily used for disk decompression (Cox, 2011). Upon achieving 50% clinical improvement and a reduction of sciatic leg pain, the patient would be transitioned to protocol 2. Protocol 2 consists of 1 set per spinal movement consisting of 10 cycles of equal distractive HF and LyAF applied in flexion, lateral flexion, and circumduction (Cox, 2011). Protocol 2 is used for disk decompression and spinal mobilization.

**Figure 1:**
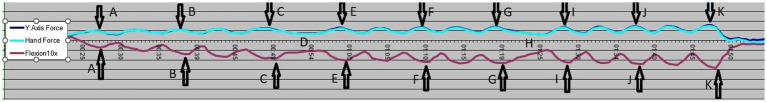
Protocol 1 graph with bi-directional forces and flexion angle values indicated.

**Figure 2:**
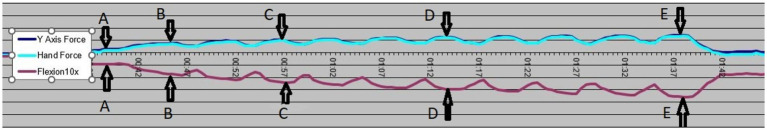
Protocol 2 - flexion graph with bi-directional forces and flexion angle values indicated.

Equal-force bi-directional traction (EqFT) used in this treatment plan is a force-based form of traction evolved from the CTFDD treatment protocols. Using a pre-set distractive distance on the C8FT, the objective for the treating doctor is to apply an equal amount of counter HF against the LyAF being generated by the C8FT, thus applying equal bi-directional traction forces. The application of EqFT is dictated by patient tolerance (Figures 1, 2 and Tables 1– 3). EqFT is a prime example of the need for interdisciplinary force-based spinal manipulation research to develop new and innovative treatment protocols, such as EqFT, to improve clinical outcomes for the treatment of low back pain (Reed et al., 2024).

**Table 1:** Treatment Abbreviations.

CTFDD	Cox Technic Flexion Distraction Decompression
EMS	Electrical Muscle Stimulation
IRL	Infra-Red-Light Therapy
EqFT	Equal Bi-Directional Force Traction

**Table 2:** Forces and flexion angle at different treatment points for protocol 1.

	Protocol 1 Values	Flexion/Hand & Long Y Forces	Flexion Angle
A	Taut Point	9.63 lbs	.71
B	3^rd^ cycle apex	11.15 lbs	1.42
C	5^th^ cycle apex	12.21 lbs	1.85
D	Pause between sets		
E	1^st^ cycle apex	12.16 lbs	2.07
F	3^rd^ cycle apex	12.93 lbs	2.19
G	5^th^ cycle apex	13.45 lbs	2.30
H	Pause between sets		
I	1^st^ cycle apex	13.32 lbs	2.24
J	3^rd^ cycle apex	13.94 lbs	2.43
K	5^th^ cycle apex	14.91 lbs	2.86

**Table 3:** Forces and flexion angles at different treatment points for protocol 2.

	Protocol 2 – Flexion	Hand & Long Y Forces	Flexion Angle
A	Taut Point	2.05 lbs	.93
B	1^st^ cycle apex	6.81 lbs	1.79
C	3^rd^ cycle apex	9.87 lbs	2.43
D	6^th^ cycle apex	12.12 lbs	2.96
E	10^th^ cycle apex	13.38 lbs	3.60

## Patient information

A 59-year-old woman sought chiropractic treatment for the management of sciatica and an inability to dorsiflex the left foot. This condition was described as having a dull, achy, burning pain at the lower left lumbar spine which radiated into the left lateral thigh and then into the left lower extremity and left foot. The left foot was described as having tingling, numbness, and weakness. This sciatica condition began 4 months prior to this initial examination and developed 2 weeks after undergoing right knee joint replacement surgery while performing a post-operative home rehabilitation program. The patient rated the pain as constant 5 of 10 on a numeric pain rating scale, with 0 representing no pain and 10 representing excruciating pain. Initially, the pain was rated at a constant 8 of 10, but it decreased to a constant 5 of 10 following prior treatment. The patient scored 26 on a low back pain (Revised Oswestry) outcome assessment. The patient described the pain as chronic (ongoing for 20 weeks) in duration and without any prior history of the present condition. The patient’s activities of daily living were limited due to low back pain and an inability to feel and dorsiflex the left foot. The patient had difficulty lifting weights, walking, sitting, standing, and traveling. The initial treatment for this sciatica condition was rendered by the orthopedic surgeon who performed the right knee replacement surgery. This treatment consisted of prescribing prednisone and a referral for physical rehabilitation. Physical rehabilitation consisted of stretching, cardiovascular exercise, and a core strengthening program. Subsequently, the patient presented to a pain management physician, who administered a series of epidural injections, and then to a neurosurgeon, who recommended spinal decompression surgery. Chiropractic treatment using HVLA spinal manipulation was also rendered. This form of spinal manipulation showed no improvement, so this chiropractor referred the patient for force-based CTFDD spinal mobilization. The patient’s past health history includes hypothyroidism, type II diabetes, and right knee joint replacement surgery (Tables 1, 4).

**Table 4:** Chronological order of prior treatment.

1	The patient underwent knee replacement surgery from an orthopedic surgeon.
2	The patient initiated physical rehabilitation at home.
3	The patient developed low back pain with sciatica 2 weeks after right knee replacement surgery.
4	The orthopedic surgeon performed the joint replacement surgery, prescribed prednisone, and referred the patient for spinal physical rehabilitation.
5	The patient commenced physical rehabilitation for sciatica.
6	The patient initiated chiropractic treatment and received HVLA manipulation. The patient did not get relief from this intervention.
7	The orthopedic surgeon prescribed additional medication and referred the patient for pain management and for a lumbar spine MRI study.
8	Pain management included three epidural injections. The patient was referred for neurosurgical consultation. The neurosurgeon recommended spinal decompression surgery.
9	The patient was referred by the chiropractor to receive Cox Technic flexion distraction spinal manipulation treatment and the condition improved.
10	The patient returned to the neurosurgeon and elected not to undergo spinal decompression surgery.
11	The patient was referred by the pain management physician for an electro-diagnostic study (EMG), which was reported as a normal EMG.

## Clinical findings

**Figure 3:**
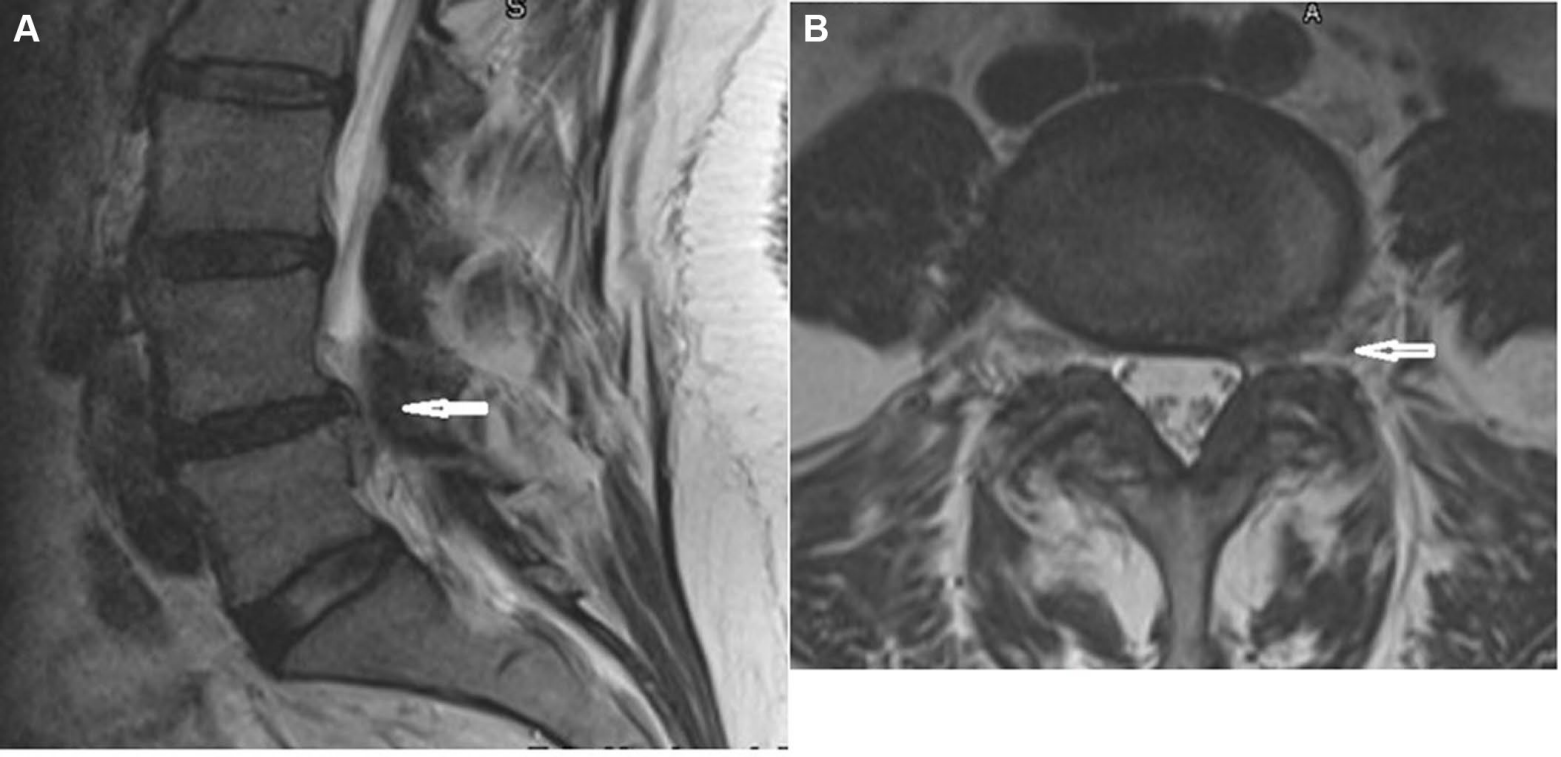
MRI of lumbar spine. **(A)** Sagittal view. **(B)** Axial view L4–L5.

The patient is 5′5″ tall, weighs 190 pounds, and demonstrated a limping gait due to numbness and weakness in the left foot. The lumbar spine ranges of motion were measured with a digital goniometer: right lateral bending at 20° with pain, left lateral bending at 20° with pain, and extension at 15° with pain. Flexion measured at 75° with mild pain. An orthopedic examination demonstrated the following positive tests: Bechterew’s test (left leg), SLR test (left leg at 35°), Kemp’s test (bilaterally), Nachlas’ test (left), and prone lumbar flexion (bilateral). These findings suggest a disk lesion, large disk herniation, or nerve root compression. Additionally, Braggard’s test (left leg) and medial hip rotation (left leg) both suggest sciatica. Manual muscle strength testing of the lower extremities, which included plantar flexion, hallux flexion, gluteus maximus, biceps femoris, and quadriceps femoris, were within normal limits and graded at 5/5 bilaterally. Dorsiflexion and hallux extension of the right foot were within normal limits and graded at 5/5. Dorsiflexion and hallux extension of the left foot were reduced and graded at 1/5. Deep tendon reflexes of the lower extremity revealed that the patellar reflexes were rated +2 bilaterally, while the Achilles reflexes were rated +1 bilaterally. Muscle spasms and tenderness were noted upon palpation of the left lumbar para-spinal musculature (L4 and L5 levels), left gluteus maximus, left piriformis muscle, left hamstring musculature, and the left popliteal fossa. The patient’s MRI images (Figure 3) showed no disk bulge or herniation, and no significant central canal or foraminal stenosis was found at the L1-L2, L2-L3, L3-L4, and L5-S1 levels. The L4-L5 level demonstrates disk bulging with a left foraminal disk herniation. A free fragment of disk material is noted at the posterior superior L5 vertebral body. The left ligamentum flavum shows hypertrophy compared to the contralateral side. Mild bilateral facet osteoarthritis is seen, with a trace of effusion at the right facet joint.

## Diagnostic assessment

Based on the subjective complaints of this patient, objective clinical findings from the orthopedic and neurological examinations, and a positive lumbar spine MRI study, a diagnosis of discogenic sciatica with a sequestered disk fragment at L5 and a motor deficit in the left lower extremity was rendered.

## Therapeutic intervention

**Figure 4:**
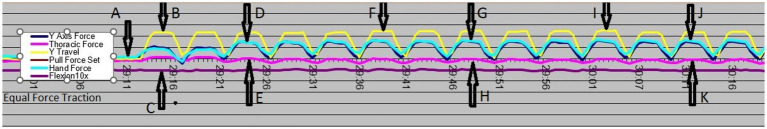
Bi-directional equal force traction graph with bi-directional forces and flexion angle values indicated.

A treatment plan consisting of force-based CTFDD spinal manipulation and EqFT for 1 min 45 s was used as a per-manipulative treatment to reduce spinal stiffness (Jun et al., 2020) (Figures 1, 2), pre-modulated electrical muscle stimulation (EMS) was applied for 7 min to the affected area to provide temporary pain relief, and 12 joules of infrared light therapy (ILT) were administered at the L4–L5 level to potentially reduce inflammation (Tables 2, 5). A home exercise program consisting of a stretching and strengthening program targeting the lumbar spine and left foot dorsiflexion, ice applications, and lifestyle modifications restricting bending and lifting was also instituted. A treatment schedule of three treatments per week for 4 weeks was initiated with the expectation of achieving 50% subjective and objective clinical improvement. Upon achieving 50% clinical improvement and reduced sciatica pain, the treatment schedule would be reduced until achieving maximum medical improvement. Subjective improvement would be measured by low back pain (Oswestry Revised) outcome assessment form and the numeric pain rating scale. The objective clinical improvement would be measured by an orthopedic/neurological re-examination using measured lumbar ranges of motion, a straight leg raise test, Braggard’s test, Kemp’s test, and manual muscle strength testing of left foot dorsiflexion. Left foot dorsiflexion will be tested at every visit to monitor muscle strength improvement. To apply force-based CTFDD spinal manipulation, a C8FT was used. The C8FT (Model #s – 16201700, 16501702, 16201699, Haven Innovations, Michigan, United States) has sophisticated force cell technology built within its cushions. These force cells are configured to accurately record the treatment forces in pounds applied during a CTFDD spinal manipulation. Additionally, the movements of the caudal (lower) section of the table of flexion and lateral flexion are recorded in degrees. Using the C8FT gives the advantage of acquiring objective treatment forces and motion data ensuring accuracy, consistency, and reproducibility of the CTFDD spinal manipulation (Gudavalli and Cox, 2014). The uniqueness of CTFDD spinal manipulation is the application of equal bi-directional forces of cephalad HF and caudalward long y axial force (LyAF) (Cox, 2011). Initially, the patient was tolerance tested for treatment viability, and then CTFDD protocol 1 was applied to this patient’s lumbar spine to treat sciatica (LyAF) (Cox, 2011) (Figures 1, 2). Upon achieving 50% clinical improvement and a reduction of sciatic leg pain, the patient was transitioned to protocol 2 (Figure 4 and Table 6) (Cox, 2011).

**Table 5:** Treatment schedule for CTFDD spinal manipulation treatment plan.

	Prior treatment (see Table 4)		
Visit	Evaluation	Treatment	Assessment
Visits 1–2	Initial evaluation: Pain rating 5/10 Muscle test 1/5	CTFDD, EMS, IRL	Initial exam, condition unchanged
Visit 3	Pain rating 4/10 Muscle test 2/5	CTFDD, EMS, IRL	20% improvement
Visits 4–7	Pain rating 4/10 Muscle test 2/5	CTFDD, EMS, IRL, Home Program	20% improvement maintained
Visit 8	Pain rating 3/10 Muscle test 3/5	CTFDD, EMS, IRL	40% improvement
Visits 9–14	Pain rating 3/10 Muscle testing 3/5	CTFDD, EMS, IRL, EqFT	40% improvement maintained
Visit 15	Pain rating 2/10 Muscle testing 4/5	CTFDD, EMS, IRL, EqFT	80% improvement
Visits 16–20	Pain rating 2/10 Muscle testing 4/5	CTFDD, EMS, IRL, EqFT	80% improvement maintained
Visits 21–22	Pain rating 1/10 Muscle testing 5/5	CTFDD, EMS, IRL, EqFT	100% improvement
	Patient released from care		

**Table 6:** Hand and longitudinal forces at different treatment points while delivering equal bi-directional treatment forces.

	Bi-Directional Traction Forces	(HF) Hand Force	(LyAF) Long Y Axial Force
A	Preload Forces	1.90 lbs	0.42 lbs
B	1^st^ cycle Travel (distraction distance) - 23.0mm		
C	Flexion Angle - 1.03°		
D	3^rd^ cycle apex forces	15.08 lbs	15.08 lbs
E	Force Differential between HF & LyAF = 0.0		
F	6^th^ cycle apex travel = 24.5mm		
G	8^th^ cycle apex forces	15.22 lbs	14.92 lbs
H	Force Differential between HF & LyAF= 0.3 lbs		
I	11^th^ cycle apex travel = 23.6 mm		
J	13^th^ cycle apex	15.44 lbs	15.14 lbs
K	Force Differential between HF & LyAF = 0.3 lbs		

## Follow-up and outcomes

The patient responded well to this treatment plan, and after 7 treatments, the pain was rated at 3/10, and dorsiflexion and hallux extension were rated at 3/5. Lumbar spine ranges of motion had increased by 10% with reduced pain; Kemp’s test was positive on the left side only, and the left leg SLR was positive at 65°. After 22 treatments, the patient rated the pain at 1/10 on a numeric pain rating scale with a low back pain (Revised Oswestry) assessment score of 8. Manuel’s muscle strength testing for dorsiflexion was rated at 5/5 bilaterally, the patient could heel and toe walk normally. The lumbar spine ranges of motion were measured: flexion at 100° without pain, extension at 20° without pain, and right and left lateral bending at 25° without pain, bilaterally. Kemp’s test was negative bilaterally, and SLR was negative at 80°, bilaterally. The patient reported improvements in her ability to lift weight, walk, sit, stand, sleep, and travel and in her social life. Subsequently, the pain management physician referred the patient for an electro-neurodiagnostic study (EMG) after the CTFDD treatment plan was completed. This post-treatment EMG study was reported as normal.

## Discussion

Discogenic sciatica is frequently caused by an L4–L5 herniated disk putting mechanical pressure on the L5 nerve root (Meng et al., 2022). As this condition progresses, weakness of the tibialis anterior, extensor digitorum, halluces longus, and extensor hallucis muscles with dysesthesia of the L5 dermatome can manifest clinically. Peroneus longus and brevis muscle weakness can also occur when an L5-S1 disk herniation compresses the S1 nerve root. Foot and great toe dorsiflexion strengths will depend on the nerve supply of the peroneal nerve to the anterior tibialis and extensor muscles.

Clinically, the straight leg raise test will be positive in proportion to the amount of nerve root compression by the intervertebral disk. Patients with only leg pain and those with a marked predominance of leg pain over back pain have a high probability of harboring an extruded disk fragment (Pople and Griffith, 1994; Cox, 2011; Niazi et al., 2020). A patient with a clinical presentation of acute sciatica in the lower extremity due to a lumbar disk herniation and sequestered fragment is commonly referred for surgical consultation and possible intervention (Wielechowski et al., 2020); however, non-surgical, conservative treatment for radicular pain may be comparable to surgery based on cost, morbidity, and complications (Neault, 1992; Ikeda et al., 1996; Komori, 1996; Memmo et al., 2000; Peul et al., 2007, 2008; Lillie, 2010; Hong and Ball, 2016). Research has shown that non-surgical treatment outcomes are equal to surgery for patients who were surgical candidates with a herniated lumbar disk and radicular pain (Neault, 1992; Ikeda et al., 1996; Komori, 1996; Peul et al., 2007, 2008; Lillie, 2010; Cox, 2011; Hong and Ball, 2016).

Cox flexion distraction spinal manipulation has specific standards of care algorithms for the efficacious management of patients with discogenic sciatica (Cox et al., 1996; Cox, 2011). Randomized clinical studies have demonstrated an increase in lumbar spine ranges of motion in low back pain patients receiving decompression therapy. Our CTFDD treatment data demonstrate a gradual increase in table motion and applied manipulative forces as the number of distraction cycles increases. This increase in manipulative forces and spinal motion post-CTFDD spinal manipulation may indicate increased lumbar spine flexibility and biomechanical improvement. Achieving demonstrable biomechanical improvement graphs from a CTFDD spinal manipulation is a possible direction for force-based spinal manipulation research (Isner-Horobeti et al., 2016; Amjad et al., 2022; Reed et al., 2024).

## Conclusion

Force-based CTFDD spinal manipulation, along with other modalities consisting of pre-modulated EMS, ILT, and a home stretching and strengthening program, has been found to be an alternative, non-surgical treatment for discogenic sciatica. An interdisciplinary approach to research on the force-based CTFDD spinal manipulation is needed to further evaluate the post-treatment phenomenon of the manipulative forces and motion applied to the lumbar spine using multiple patients and/or a randomized clinical study.

## Data availability statement

The raw data supporting the conclusions of this article will be made available by the authors, without undue reservation.

## Ethics statement

The studies involving humans were approved by Keiser University Institutional Review Board. The studies were conducted in accordance with the local legislation and institutional requirements. The participants provided their written informed consent to participate in this study. Written informed consent was obtained from the individual(s) for the publication of any potentially identifiable images or data included in this article.

## Author contributions

TS: Writing – review & editing, Writing – original draft, Investigation, Conceptualization. MG: Writing – review & editing, Writing – original draft, Methodology, Conceptualization. RK: Writing – review & editing, Writing – original draft.
